# Assessment of multiple mycotoxin exposure and its association with food consumption: a human biomonitoring study in a pregnant cohort in rural Bangladesh

**DOI:** 10.1007/s00204-022-03288-0

**Published:** 2022-04-19

**Authors:** Nicholas N. A. Kyei, Benedikt Cramer, Hans-Ulrich Humpf, Gisela H. Degen, Nurshad Ali, Sabine Gabrysch

**Affiliations:** 1 Institute of Public Health, Charité–Universitätsmedizin Berlin, corporate member of Freie Universität Berlin and Humboldt-Universität zu Berlin, Charitéplatz 1, 10117 Berlin, Germany; 2 Heidelberg Institute of Global Health, Heidelberg University, Im Neuenheimer Feld 324, 69120 Heidelberg, Germany; 3 Research Department 2, Potsdam Institute for Climate Impact Research (PIK), Member of the Leibniz Association, P. O. Box 60 12 03, 14412 Potsdam, Germany; 4 Institute of Food Chemistry, Westfälische Wilhelms-Universität Münster, Corrensstr. 45, 48149 Münster, Germany; 5 Leibniz-Research Centre for Working Environment and Human Factors (IfADo) at the TU Dortmund, Ardeystr. 67, 44139 Dortmund, Germany; 6 Department of Biochemistry and Molecular Biology, Shahjalal University of Science and Technology, Sylhet, Bangladesh

**Keywords:** Mycotoxins, Human biomonitoring, Exposure assessment, Pregnant women, Risk assessment, Rural areas, Urine

## Abstract

Aflatoxins (AFs), ochratoxin A (OTA), citrinin (CIT), fumonisin B_1_ (FB_1_), zearalenone (ZEN), and deoxynivalenol (DON) are mycotoxins that may contaminate diets, especially in low-income settings, with potentially severe health consequences. This study investigates the exposure of 439 pregnant women in rural Bangladesh to 35 mycotoxins and their corresponding health risks and links their exposure to certain foods and local stimulants. Overall, 447 first-morning urine samples were collected from pregnant women between July 2018 and November 2019. Mycotoxin biomarkers were quantified by DaS-HPLC–MS/MS. Urinary concentration of frequently occurring mycotoxins was used to estimate dietary mycotoxin exposure. Median regression analyses were performed to investigate the association between the consumption of certain foods and local stimulants, and urinary concentration of frequently occurring mycotoxins. Only in 17 of 447 urine samples (4%) were none of the investigated mycotoxins detected. Biomarkers for six major mycotoxins (AFs, CIT, DON, FB_1_, OTA, and ZEN) were detected in the urine samples. OTA (95%), CIT (61%), and DON (6%) were most frequently detected, with multiple mycotoxins co-occurring in 281/447 (63%) of urine samples. Under the lowest exposure scenario, dietary exposure to OTA, CIT, and DON was of public health concern in 95%, 16%, and 1% of the pregnant women, respectively. Consumption of specific foods and local stimulants—betel nut, betel leaf, and chewing tobacco—were associated with OTA, CIT, and DON urine levels. In conclusion, exposure to multiple mycotoxins during early pregnancy is widespread in this rural community and represents a potential health risk for mothers and their offspring.

**Supplementary Information:**

The online version contains supplementary material available at 10.1007/s00204-022-03288-0.

## Introduction

Mycotoxins are natural toxins produced as secondary metabolites of micro-fungi under certain environmental and crop conditions; they are harmful to humans and animals even in low concentrations (Bennett and Klich 2003; Bhatnagar et al. 2002). Mycotoxins contaminate a substantial proportion of a variety of dietary staples and other food crops worldwide (Eskola et al. 2020). Due to their resistance to standard food processing and cooking practices, mycotoxins remain an important dietary contaminant, especially in low-income countries (IARC 2015), with potentially serious health consequences (Marin et al. 2013; Turner et al. 2012).

Human exposure to mycotoxins can either be assessed through environmental monitoring or biological monitoring (biomonitoring). Environmental monitoring involves directly measuring mycotoxin levels in food, feed, and/or air. However, representative mycotoxin contamination data from food are difficult to obtain due to the high degree of heterogeneity of food, the possible presence of modified forms of mycotoxins (Rychlik et al. 2014) and, in many regions, the challenges in sampling foods produced by subsistence farmers (IARC 2015). On the other hand, biomonitoring involves detecting mycotoxins, mycotoxin metabolites, and adducts (referred to as biomarkers) in tissues, body fluids, and excreta. Current state-of-the-art biomarker analysis methods enable relatively easy and objective detection and quantification of major mycotoxins from bio-samples (Escrivá et al. 2017; Ezekiel et al. 2022; Šarkanj et al. 2018). Examples of major mycotoxins investigated include aflatoxins (AFs), fumonisin B1 (FB1), ochratoxin A (OTA), citrinin (CIT), zearalenone (ZEN), and deoxynivalenol (DON) (Al-Jaal et al. 2019; Gerding et al. 2015, 2014; Schmidt et al. 2021a, 2021b; Solfrizzo et al. 2014; Warth et al. 2014). Biomonitoring of exposure biomarkers is also considered advantageous, as it provides information on the internal dose of exposure via all routes, including dietary intake and exposures via the respiratory tract and the skin (Degen 2011; Fromme et al. 2016). Moreover, biomonitoring is particularly valuable in low-income settings, such as Bangladesh, where regular food contamination surveillance is scarce or non-existent (Islam and Hoque 2013).

Human biomonitoring studies from several countries demonstrate widespread exposure to major mycotoxins during pregnancy (Abdulrazzaq et al. 2002; Chan-Hon-Tong et al. 2013; Groopman et al. 2014; Jonsyn et al. 1995; Lei et al. 2013). There is also evidence of transplacental transfer of mycotoxins such as AFs, OTA, CIT, DON, and ZEN to the developing fetus in humans and animals (Goyarts et al. 2007; Nielsen et al. 2011; Partanen et al. 2010; Reddy et al. 1982; Warth et al. 2019; Woo et al. 2012). This suggests a potentially harmful exposure during the critical first 1000 days of a child's life (Groopman et al. 2014; Ismail et al. 2021), with some evidence of adverse effects on newborn and child health (Andrews-Trevino et al. 2021, 2019; Kyei et al. 2020; Sherif et al. 2009).

Bangladesh has a tropical monsoon climate, characterized by seasonal variations in rainfall, high temperatures, and high humidity, conducive for mold contamination of food crops and subsequent mycotoxin production. An environmental monitoring study in the early 1990s assessed the extent of mycotoxin contamination of Bangladeshi food groups, including cereals, nuts, and pulses, in four districts (Chittagong, Dhaka, Rajshahi, and Khulna). In that study, varying contamination levels of major mycotoxins were detected in food samples, including AFs, FB_1_, DON, OTA, and ZEN (Dawlatana et al. 2002). Similarly, in a more recent market study, eight common Bangladeshi foods, including rice, wheat flour, lentils, groundnut, and spices, as well as betelnut were sampled from three cities: Dhaka, Chittagong, and Sirajganj, and analyzed for aflatoxin contamination. In that study, high levels of aflatoxins were detected in dates, groundnut, betelnut, and red chili powder (Roy et al. 2013). A very recent study reported the presence of aflatoxin M_1_ (AFM_1_) in dairy milk and milk products collected in four major divisions in Bangladesh (Sumon et al. 2021).

Consistent with these limited environmental monitoring studies, the results from biomonitoring studies in Bangladesh also suggest dietary exposure to mycotoxins for different population groups. For example, several biomonitoring studies analyzing urine samples from adults in rural and urban areas in the Rajshahi district reported the frequent occurrence of major mycotoxins such as AFM_1_, OTA, CIT, ZEN, and DON (Ali et al. 2015b, 2016a, b, d, 2017; Ali and Degen 2019). Studies analyzing urine samples from pregnant women in Dhaka district also reported the frequent occurrence of AFM_1_, OTA, CIT, and DON (Ali et al. 2015a, 2016c, 2017). Two other recent studies that analyzed urine samples from young children in Rajshahi and Dhaka districts also report a frequent occurrence of AFM_1_, DON, OTA, and CIT (Ali and Degen 2020; Ali et al. 2020). Furthermore, a recent study indicated the presence of AFM_1_ in about half of breast milk samples in Sylhet division (Islam et al. 2021).

Most biomonitoring studies have focused on characterizing individual mycotoxin occurrences rather than characterizing concurrent exposures to multiple mycotoxins. However, with molds requiring similar toxin production conditions and different mold species contaminating a food sample, concurrent exposure to multiple mycotoxins is likely to occur in reality (Frisvad et al. 2011; Turner et al. 2012; Vidal et al. 2018). Consequently, to improve the understanding of the mechanisms behind the association between maternal exposures to mycotoxins and adverse pregnancy outcomes in a population, there is a need to determine and describe the extent of simultaneous exposure of pregnant women to different mycotoxins.

Our study was conducted amongst a cohort of pregnant women in rural Habiganj district, Sylhet division, Bangladesh, where no previous environmental or biomonitoring studies for mycotoxins have been reported. The study aims to: (i) describe the occurrence and types of mycotoxin exposure of pregnant women in rural Bangladesh, (ii) estimate the probable daily intake (PDI) levels of frequently occurring mycotoxins and estimate dietary exposure of public health concern, and (iii) explore possible associations between the consumption of common Bangladeshi foods and stimulants and urinary concentrations of frequently detected mycotoxins.

## Materials and methods

### Study population and design

The *Food and Agricultural Approaches to Reducing Malnutrition* (FAARM) cluster-randomized controlled trial was conducted in two sub-districts of Habiganj district in Bangladesh’s Sylhet division (ClinicalTrials.gov ID: NCT02505711). FAARM included 2700 young married women, with self-reported age below 30 years, in 96 settlements (geographic clusters) who reported an interest in gardening and had access to at least 40 m^2^ of land. Settlements were randomized into 48 intervention and 48 control clusters. The FAARM trial evaluates the impact of a homestead food production program, implemented by the international non-governmental organization Helen Keller International, on undernutrition in young children. Further information on the FAARM trial design is available in the study protocol (Wendt et al. 2019).

As an add-on to the FAARM trial, we conducted a prospective cohort study: *Maternal Exposure to Mycotoxins and Adverse Pregnancy Outcomes* (MEMAPO). MEMAPO enrolled a subsample of pregnant FAARM women—recruited early in pregnancy, of which 439 were followed up until the end of pregnancy—to investigate the role of maternal exposure to mycotoxins for the development of specific adverse pregnancy outcomes.

### Urine sample collection and processing

We obtained 447 urine samples from 439 pregnant women (8 women with two pregnancies) who consented to participate in the MEMAPO study. About 10 ml of first-morning urine sample was collected into a sterile disposable container from each study participant before consuming food and water that day. The urine samples were collected between July 2018 and November 2019. They were transported to the project field laboratory in a cold box, aliquoted into 2 ml safe-seal tubes, and stored at  – 20 °C on the same day of urine pick-up. All samples were subsequently sent to Germany on dry ice (transport time < 24 h) and stored at  – 70 °C at Heidelberg University. Samples were later transported on dry ice to Münster University, Germany (transport time < 6 h) and stored at  – 80 °C until biomarker analysis.

### Background characteristics of study participants

Data on descriptive characteristics, such as a woman’s religion, her highest level of education, and her height, were extracted from the FAARM baseline and endline surveys conducted in 2014 and 2019, respectively. Using the FAARM endline survey's asset module, the household’s relative position within the 2014 Demographic and Health Survey (DHS) national wealth quintiles was calculated using the Equity Tool (NIPORT et al. 2016; Metrics for Management 2016). Data on other quantitative descriptive characteristics, such as gestational age in weeks and current weight, were extracted from the FAARM routine surveillance round at the time when the woman was enrolled in MEMAPO. The woman’s age at pregnancy enrollment was computed from her age at the FAARM baseline survey.

### Food consumption data

FAARM’s routine surveillance system collects information on women’s dietary diversity, using a 21-food group questionnaire which can be collapsed into ten food groups according to recommended guidelines (FAO and FHI 360 2016). Briefly, women are asked about the food they consumed the previous 24-h day through an open recall followed by list-based probes. When women reported consuming a food item, they were then asked if they had consumed more or less than a spoonful to assess whether they had consumed at least 15 g of the food item throughout the day. Responses are aggregated into food groups, including starches (e.g., grains and white roots or tubers), pulses, nuts and seeds, dairy, flesh foods (e.g., meat, poultry, and game), fish, organ meats, eggs, dark green leafy vegetables, vitamin A-rich fruits and vegetables, other fruits, other vegetables, and sugary foods (sugar, honey, juice, chocolates, etc.). In the FAARM surveys, women were also asked about their intake of common local stimulants such as betelnut, betel leaf (locally called *Paan*), and chewing tobacco (locally called *Jorda*) in the past month.

### Urine analysis

#### Urine creatinine and urine density analysis

Standard routine urine analyses were performed in the accredited central laboratory of Heidelberg University Hospital. Routine urine status parameters including specific gravity were assessed via multi-parameter urine test strips (#10,634,643, Siemens Healthcare GmbH, Erlangen, Germany) on a Siemens CLINITEK Novus® analyzer according to the manufacturer’s instructions. Urine creatinine concentrations were quantified on a Siemens ADVIA XPT chemistry analyzer (Siemens kit #03,039,070).

#### Mycotoxin analysis in spot urine

Mycotoxin biomarkers in urine were quantified by a validated “dilute and shoot” method using high-performance liquid chromatography–tandem mass spectrometry detection (DaS-HPLC–MS/MS), according to Gerding et al. (2014). This method was extended to include 12 additional analytes, so that urine samples were screened for 35 biomarkers in a chromatographic run time of 13 min. Instrumental limits of all analytes in urine were determined in a range of 0.01 to 30 ng/ml for the limit of detection (LOD) and 0.03 to 100 ng/ml for the limit of quantification (LOQ) (Table 1).

**Table 1:** Instrumental limits of mycotoxin biomarker concentrations in urine in ng/ml

35 Mycotoxin biomarkers	LOD	LOQ
Aflatoxin B_1_ (AFB_1_)	0.06	0.2
Aflatoxin B_2_ (AFB_2_)	0.04	0.12
Aflatoxin M_1_ (AFM_1_)	0.1	0.3
Aflatoxin G_1_ (AFG_1_)	0.7	2
Aflatoxin G_2_ (AFG_2_)	0.2	0.6
Alternariol (AOH)	3	10
Alternariol monomethyl ether (AME)	0.3	1
Altenuene (ALT)	1.7	5
Beauvericin (BEA)	1	2.5
Citrinin (CIT)	0.17	0.5
Deoxynivalenol (DON)	1.7	5
Deoxynivalenol-3-glucuronide (DON-3-GlcA)	2.5	7.5
Deoxynivalenol-15-glucuronide (DON-15-GlcA)	2.5	7.5
Dihydrocitrinone (HO-CIT)	0.1	0.3
Enniatin B (ENB)	0.025	0.075
Enniatin B_1_ (ENB_1_)	0.125	0.375
Enniatin A (ENA)	0.06	0.175
Enniatin A_1_ (ENA_1_)	0.12	0.35
Fumonisin B_1_ (FB_1_)	1	3
Fumonisin B_2_ (FB_2_)	30	100
HT2-toxin (HT2)	8	25
HT2-toxin-3-glucuronide (HT-2–3-GlcA)	1.7	5
HT2-toxin-4-glucuronide (HT-2–4-GlcA)	1.3	4
Hydroxy-Ochratoxin A (OH-OTA)	0.05	0.15
Ochratoxin A (OTA)	0.02	0.06
2'R-Ochratoxin A (2'R-OTA)	0.01	0.03
Ochratoxin alpha (OTalpha)	0.2	0.6
T2-toxin (T2)	0.2	0.6
Zearalenone (ZEN)	0.7	2
Zearalenone (ZAN)	1.25	3.75
Zearalanone-14-glucuronide (ZAN-14-GlcA)	7	20
Zearalenone-14-glucuronide (ZEN-14-GlcA)	7	20
alpha-Zearalenol-14-glucuronide (alpha-ZEL-14)	3	10
beta-Zearalenol-14-glucuronide (beta-ZEL-14)	7	20
Zearalenone-14-sulfate (ZEN-14-SO_4_)	0.3	1

*LOD* limit of detection, *LOQ* limit of quantification

### Statistical analysis

#### Management of left censoring and descriptive analysis

Left-censored urine mycotoxin data, i.e., concentrations below the LOD and LOQ of the analytical method, were imputed using the substitution methods as recommended in the European Food Safety Authority (EFSA) guidelines (EFSA 2010). Following EFSA guidelines, three substitution scenarios were estimated, lower bound (LB), middle bound (MB), and upper bound (UB). Specifically, results below LOD were substituted with the value of zero, LOD/2, and LOD under LB, MB, and UB scenarios, respectively. Likewise, results below LOQ were substituted with the value of LOD, LOQ/2, and LOQ under LB, MB, and UB scenarios, respectively. To adjust for inter-individual variation in urine volume, urine mycotoxin concentrations were corrected for urine density and urine creatinine before dietary exposure estimation. Descriptive statistics, including mean urine concentration of mycotoxins, standard deviation, and concentration range, were thus calculated for all substitution scenarios, corrected for urine density and creatinine concentration (Supplementary Table S1). Although urine density-corrected concentrations are preferred (Carrieri et al. 2001; Suwazono et al. 2005), both an adjustment for density and for creatinine are presented for easy comparison with previous studies using either method. For density correction, the formula provided by Smith et al. (2012) was adopted:

$$C\left( {cor} \right) = \left[ {C\left( {obs} \right)*\left( {1.0104 - 1} \right)} \right]/\left( {\rho - 1} \right)],$$

where *C*(cor) is the adjusted concentration in ng/ml, *C*(obs) is the observed unadjusted concentration in ng/ml, 1.0104 is the average urine density in our dataset (*n* = 447), and ρ is the specific density in each urine sample.

#### Estimation of dietary mycotoxin exposure

Dietary exposure assessments were performed by estimating probable daily intake (PDI) for the frequently occurring mycotoxins (OTA, CIT, DON, and AFM_1_) in this population. For DON and CIT, where both parent compounds and metabolites were detected, exposure assessment calculations were performed on total DON (DON + DON-15GlcA + DON-3GlcA) and total CIT (CIT + Dihydrocitrinone (HO-CIT)) by converting metabolites using their molar mass. The urinary mycotoxin concentrations were used to calculate a probable daily intake (PDI) in ng/kg body weight (bw) for each individual using this formula (Solfrizzo et al. 2014):

$$PDI \left( {\frac{ng}{{kg}}bw} \right) = \left[ {C*\frac{V}{W*E}} \right]*100,$$

where *C* is the mycotoxin concentration, adjusted for urine density or creatinine, *W* is the individual's body weight (kg), *E* is the excretion rate (%) of the mycotoxin, and *V* is the daily urine excretion volume (ml). A mean daily urine output during pregnancy of 2000 ml was used based on literature (Mikhail and Anyaegbunam 1995). The urinary excretion rate of DON used for the calculation was 68% (Warth et al. 2013), a value used in previous studies in Bangladesh (Ali et al. 2015a, 2016b; Gerding et al. 2015). For CIT, an excretion rate of 40.2% was used (Degen et al. 2018), while an excretion rate of 1.5% was used for AFM_1_ (Zhu et al. 1987). An excretion rate of 2.6% was adopted for OTA, based on a recent pig study (Gambacorta et al. 2019). This value is close to what was estimated from a human kinetic study (Degen 2015; Studer-Rohr et al. 2000). PDIs for the mean population mycotoxin concentrations were calculated for the LB, MB, and UB substitution scenarios.

#### Health risk characterization

Two different approaches were used for risk assessment of individual mycotoxins based on their toxicity profiles. Considering the carcinogenicity of OTA and AFB_1_, no level of exposure is considered safe. The Margin of Exposure (MOE) was thus calculated for these mycotoxins to help prioritize risk management actions. MOE was computed as a ratio of benchmark dose lower confidence limit (BMDL_10_) and women's exposure (estimated by PDI). The scientific committee of EFSA and the WHO consider a MOE of 10,000 or more to be of low concern for public health (EFSA 2013). BMDL_10_ values for aflatoxins of 0.4 µg/kg bw/day (EFSA CONTAM Panel (EFSA Panel on Contaminants in the Food Chain) et al. 2020b) and for OTA with neoplastic effects at 14.5 µg/kg bw/day (EFSA CONTAM Panel (EFSA Panel on Contaminants in the Food Chain) et al. 2020a) were used for MOE estimations. For the other mycotoxins, the estimated PDIs were compared with established health-based guidance values to calculate a hazard quotient (HQ), *i.e.,* the proportion of the population who consume these mycotoxins above established tolerable daily intake (TDI) levels. The TDI values for CIT of 200 ng/kg bw/day and for DON of 1000 ng/kg bw/day were those derived by scientific committees, such as the Joint FAO/WHO Expert Committee on Food Additives (JECFA) and the EFSA (EFSA 2012, 2017; FAO/WHO 2011). When the HQ was less than one, the exposure was considered to be within safe limits.

#### Assessment of the relationship between food consumption and urinary mycotoxin concentrations

Shapiro–Wilk’s test was used to assess normality of the raw and log-transformed urine concentration data for OTA, DON, CIT. There was strong evidence (*p*-values < 0.001) that the data, except for the log-transformed OTA concentration (*p* = 0.14), deviated from a normal distribution. Furthermore, household characteristics such as wealth did not appear to be associated with urinary mycotoxin concentration in our data. Also, an a priori model with wealth did not improve model fit significantly. Thus, crude bivariable median regression analyses were performed to investigate the direction of association between mycotoxin biomarker concentrations (OTA, total DON, and total CIT) and the consumption of typical Bangladeshi foods, as consumed by the women around the time of enrollment and reported in Table 5, and stimulants, including betel nut and betel leaf. We used foods from 19 out of the 21 dietary diversity food groups, leaving out the groups of other fruits and other vegetables. All median regression models used bootstrapping over 1000 replications to produce robust estimates. Analyses were performed in Stata 14.2 (Stata Corporation, College Station, TX, USA).

## Results

### Description of the study population

Of the cohort of 439 women recruited for the MEMAPO study, 8 had two pregnancy events during the study period, resulting in 447 pregnancy observations. The background characteristics of the enrolled pregnant women are presented in Table 2. Three-quarters were Muslim, and one-quarter were Hindu. Almost 80% came from households belonging to the middle and upper wealth quintiles of the 2014 Bangladesh Demographic and Health Survey wealth index (NIPORT et al. 2016), while a tenth belonged to the wealthiest quintile and another tenth to the lower two wealth quintiles. On average, women had been married in their late teens and were in their late twenties at the time of enrollment into this study. Only 6% of women had completed secondary school or higher degrees, while 14% had no formal education. On average, the women weighed 47 kg and were enrolled at around 15 weeks of gestation.

**Table 2:** Sociodemographic characteristics of study population

Characteristic	N*	*n*	Percent
**Religion**	439		
Muslim		331	75
Hindu		108	25
**Household wealth quintile (as in DHS 2014)**^**1**^	439		
Poorest		6	1
Lower		44	10
Middle		169	39
Upper		174	40
Wealthiest		46	10
**Woman's highest education level**	439		
No formal education		62	14
Partial primary		121	28
Complete primary		99	23
Partial secondary		134	31
Completed secondary		12	3
Post-secondary		11	3
**Season of enrollment and urine collection**	447		
January–April		112	25
May–August		188	42
September–December		147	33
**Continuous variables**		Mean	SD
Woman’s height (cm)	439	150	6
Woman’s age at first marriage (years)	435	18	2
Woman's age at enrollment (years)	447	27	4
Gestational age at enrollment (weeks)	447	15	6
Woman's weight at enrollment (kg)	447	47	8
Urine creatinine concentration (mg/100 ml)	447	61	42
Urine density (g/ml)	447	1.010	0.005

^1^This is not a relative wealth quintile, but the estimate of the households’ national wealth quintile if the assets owned in 2019 were in the 2014 DHS survey (constructed using https://www.equitytool.org), *SD* Standard deviation; *Differences in numbers as some variables are women characteristics and others pregnancy characteristics

### Occurrence of mycotoxin biomarkers in urine

**Fig. 1:**
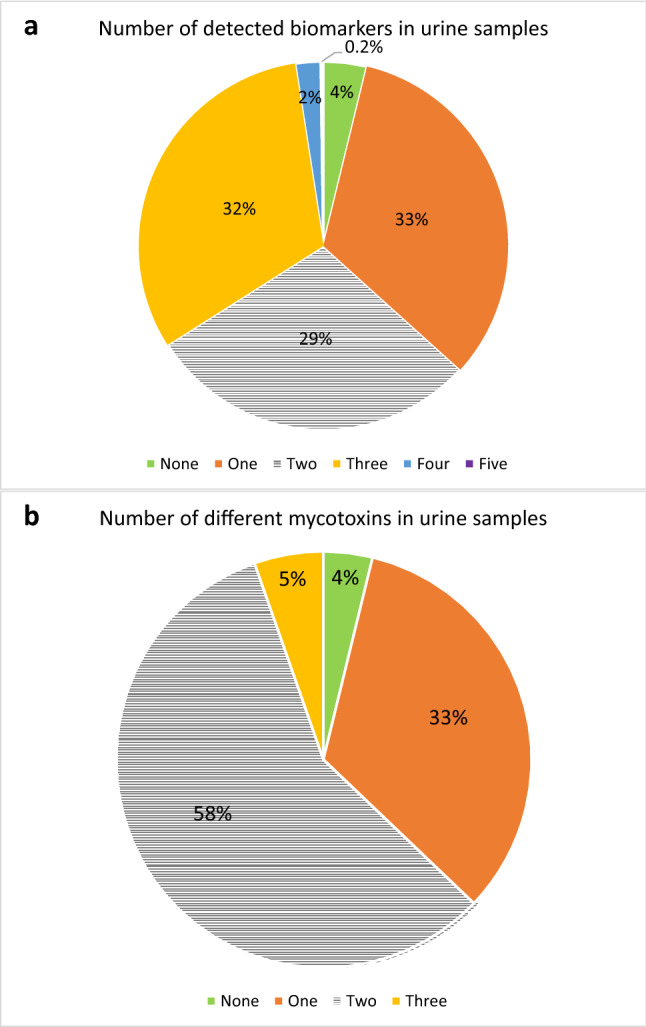
Number of detected biomarkers and mycotoxins in urine samples of pregnant women in rural Bangladesh (*n* = 447). Only in 17 urine samples (4%) none of the investigated mycotoxins were detected. About a third of the urine samples contained one, a third two, and another third three detectable mycotoxin biomarkers. Only 2% contained more than three. (Figure 1a). This corresponds to two different mycotoxins detected in over half and three in 5% of the samples, while a third of samples contained one detectable mycotoxin. (Figure 1b). tCIT- total CIT (CIT+ HO-CIT); tDON- total DON (DON + DON-Glucuronides); tAF- total aflatoxins (AFM1+AFB2)

Out of the 35 different urinary mycotoxin biomarkers investigated, ten different biomarkers were detected, representing six major mycotoxins: AF, CIT, DON, FB_1_, OTA, and ZEN (Table 3). Only 17 urine samples (4%) were free from all the investigated mycotoxin biomarkers. About a third of the urine samples each contained one, two, and three detectable mycotoxin biomarkers (Fig. 1a). More than half (58%) of the urine samples had two different detectable mycotoxins, 5% had even three detectable mycotoxins, while a third had only one detectable mycotoxin (Fig. 1b). The occurrence and uncorrected concentration of the ten detected mycotoxin biomarkers, along with their limits of detection (LOD) and quantification (LOQ) of the method, are summarized in Table 3. OTA was the most frequently occurring mycotoxin detected in 95% of the samples, with 77% of the samples detected above the LOQ of the method. The urinary metabolite of OTA, ochratoxin α (OTα), was not detected in any sample.

**Table 3:** Occurrence and concentration of mycotoxin biomarkers in urine samples of pregnant women in rural Habiganj district, Bangladesh (*n* = 447)

Mycotoxin / metabolite	Instrumental limits for biomarkers in urine (ng/ml)		Total detected ≥ LOD	Total detected > LOQ	Positive detection (%)	Uncorrected concentration in urine (ng/ml)		
	LOD	LOQ				Mean^*1*^	Min	Max
AFB_2_	0.04	0.12	1	0	< 1	–	–	–
AFM_1_	0.10	0.30	7	1	1.6	0.42	–	–
CIT	0.17	0.50	183	131	41	2.46	0.58	14.54
HO-CIT	0.10	0.30	229	82	51	1.36	0.30	10.31
*t*CIT	–	–	274	–	61			
DON	1.70	5.00	18	2	4.0	8.04	3.14	25.59
DON-3-GlcA	2.50	7.50	1	1	< 1	–	–	–
DON-15-GlcA	2.50	7.50	12	12	2.7	79.77	8.3	181.7
*t*DON	–	–	27	–	6.0			
FB_1_	1.00	3.00	1	1	< 1	9.12	–	–
OTA	0.02	0.06	424	346	95	0.32	0.06	2.93
ZEN-14-SO_4_	0.30	1.00	1	0	< 1	–	–	–

Positive samples refer to samples containing the analyte ≥ stated limit of detection (LOD). ^*1*^Only positive samples > LOQ are considered in calculating mean concentrations where applicable; *tCIT*- total CIT (CIT + HO-CIT); *t*DON- total DON (DON + DON-Glucuronides)

**Fig. 2:**
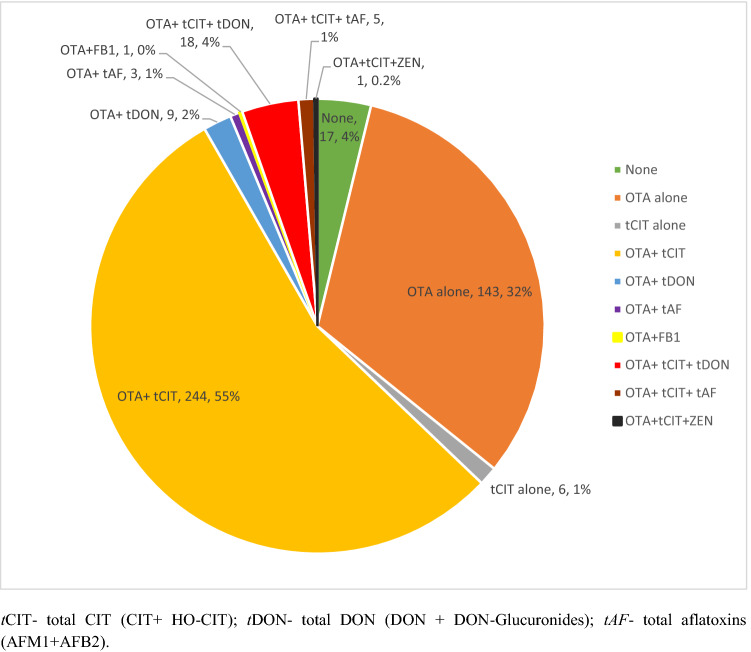
Mycotoxin combinations and their occurrence in urine samples of pregnant women in rural Bangladesh (*n* = 447). Biomarkers for six major mycotoxins (AFs, CIT, DON, FB1, OTA, and ZEN) were detected in the urine samples. Co-occurrence of multiple mycotoxins was detected in 281/447 (63%) of urine samples. Co-occurrence of OTA and CIT was the prevailing co-exposure (55%)

The next most frequently occurring mycotoxin was CIT and its metabolite HO-CIT, detected in 41% and 51% of the samples, respectively. DON and its DON-Glucuronide metabolites were detectable in 6% of samples, with no detection of de-epoxy-deoxynivalenol (DOM-1), another urinary metabolite of DON. Aflatoxins (AFB_2_ and AFM_1_) were detected in less than 2% of samples. The occurrence of different combinations of mycotoxins in the urine of pregnant women is shown in Fig. 2. Co-occurrence of OTA and CIT was the prevailing mycotoxin exposure, present amongst more than half of pregnant women (55%). About a third were exposed to OTA alone, while another 6% were exposed to CIT alone. The seven remaining detected mycotoxins always co-occurred jointly with the more frequently detected mycotoxins.

### Dietary mycotoxin exposure and health risk assessment

**Fig. 3:**
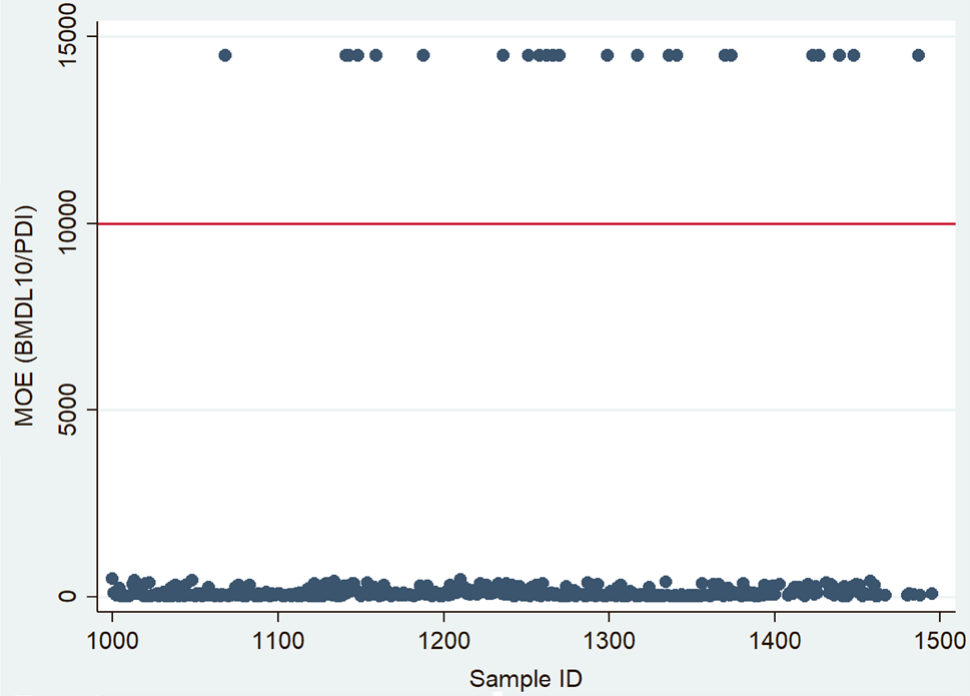
Margin of Exposure of pregnant women to dietary ochratoxin A under the lowest exposure scenario (*n* = 447). The Margin of Exposure (MOE) is calculated by dividing the benchmark dose lower confidence limit (BMDL10) for ochratoxin A (OTA; 14.5 µg/kg body weight/day) by the woman's exposure which is estimated by probable daily intake (PDI). The red line shows a MOE of 10,000. If the MOE is below 10,000, the exposure could be of health concern. Dietary exposure to OTA is below this line in 95% of pregnant women in our sample

The probable daily intake (PDI) of the three most commonly detected mycotoxins, OTA, total CIT, and total DON calculated from urine mycotoxin concentration, corrected for urine density and creatinine, and estimated for LB, MB, and UB scenarios are presented in Table 4. The estimated mean dietary exposure to OTA in the study population ranged from 400 ng/kg bw under the LB scenario to 426 ng/kg bw under the UB scenario. The maximum estimated PDI for OTA was 3968 ng/kg bw and 8070 ng/kg bw when using density-adjusted and creatinine-adjusted data. Even under the LB scenario, the Margin of Exposure (MOE) values of OTA were lower than 1000 in nearly all samples (95%), indicating a high health concern for this mycotoxin (Fig. 3).

**Table 4:** Estimated probable daily intake of four frequently occurring mycotoxins/metabolites under different substitution scenarios and with adjustment for urine density and creatinine concentration (*n* = 447)

Left-censorship substitution	Mycotoxin	PDI (ng/kg bw/day) density-adjusted				PDI (ng/kg bw/day) creatinine-adjusted			
		Mean	Standard deviation	Maximum	Health concern* *n* (%)	Mean	Standard deviation	Maximum	Health concern *n* (%)
LB	*t*CIT	116	270	2885	74 (16)	210	484	4557	111 (25)
	*t*DON	25	260	4943	3 (1)	40	373	5359	4 (1)
	OTA	400	466	3968	424 (95)	704	842	8070	424 (95)
MB	*t*CIT	131	265	2885	75 (17)	238	477	4557	116 (26)
	*t*DON	229	290	4967	4 (1)	435	572	8062	21 (5)
	OTA	407	461	3968	447 (100)	720	832	8070	447 (100)
UB	*t*CIT	152	261	2885	83 (19)	279	473	4557	149 (33)
	*t*DON	438	385	4992	20 (4)	838	968	16,123	107 (24)
	OTA	426	451	3968	447 (100)	757	816	8070	447 (100)

*PDI* probable daily intake, *eqv* equivalents, *LB* lower bound substitution, *MB* middle bound substitution, *UB* upper bound substitution, *na* not available, *Health concern- Hazard quotient > 1 for CIT and DON and Margin of Exposure < 10,000 for OTA,  Tolerable daily intake – for CIT: 200 ng/kg bw and for DON:1000 ng/kg bw, *t*CIT- total CIT (CIT + HO-CIT), *t*DON- total DON (DON + DON-Glucuronides)

**Fig. 4:**
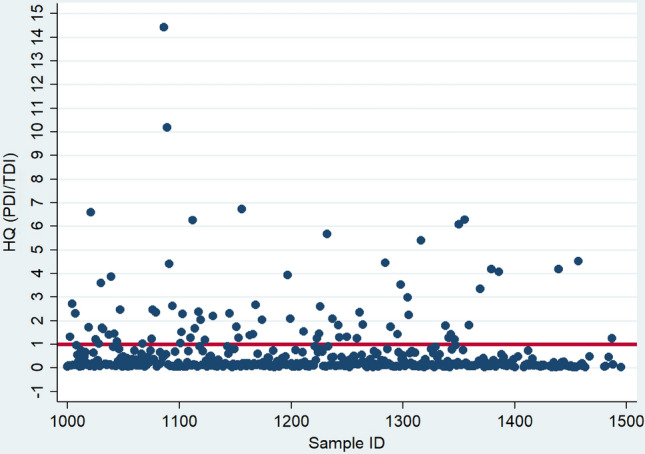
Hazard quotient of dietary exposure of pregnant women to citrinin (*n* = 447) under a moderate exposure scenario. The Hazard quotient (HQ) is calculated by dividing individual exposure, measured by probable daily intake (PDI) by the established tolerable daily intake (TDI) level for citrinin (CIT; 200 ng/kg body weight/day). The red line shows a HQ of 1. If the HQ is above 1, the exposure could be of health concern. The exposure of 17% of the pregnant women in our sample is of public health concern

The mean PDI of total CIT amongst the pregnant women using density-adjusted data ranged from 116 ng/kg bw (LB scenario) to 152 ng/kg bw (UB scenario). Compared to the established health guidance level of no concern for nephrotoxicity below 200 ng/kg bw (EFSA 2012), the exposure of 17% of the pregnant women under the MB scenario was of public health concern (Fig. 4). The proportion of pregnant women with an estimated mean dietary intake of CIT exceeding this health reference value ranged from 16% (LB scenario) to 19% (UB scenario). Using creatinine-adjusted data, the proportion of pregnant women with average intake above the health-based guidance value ranges between 25% (LB scenario) and 33% (UB scenario).

The mean PDI of total DON exceeded the group tolerable daily intake (TDI) for DON (EFSA 2017; FAO/WHO 2011) of 1000 ng/kg bw in approximately 1% (*n* = 3, density-adjusted, and *n* = 2, creatinine-adjusted data) of the participants under the LB scenario. The proportion of women with PDIs exceeding the health-based guidance value increases to 4% (*n* = 20, density-adjusted data) and 24% (*n* = 107, creatinine-adjusted data) under the UB scenario. The maximum estimated individual PDI for total DON ranges from 4943 ng/kg bw (LB scenario) to 4992 ng/kg bw (UB scenario). The corresponding maximum intake of total DON using creatinine-adjusted data ranges from 5359 ng/kg bw (LB scenario) to 16,123 ng/kg bw (UB scenario). Thus, these maximum intake values range between more than 50% to twice the acute reference dose (ARfD) of 8000 ng/kg bw established by the EFSA.

### Relationship between food consumption and mycotoxin concentration in urine

Out﻿﻿ of the 447 individual pregnancy events, four women had missing information on their food consumption around the time of recruitment. Hence, 443 pregnancy events with food consumption were used in this analysis. The number of women who consumed the specific food groups along with results of median regression analyses evaluating the direction of crude associations between concentrations of OTA, total CIT, and total DON in urine under the MB scenario and the consumption of specific Bangladeshi foods, including local stimulants, are shown in Table 5. All women consumed starchy staples such as grains and white roots in similar amounts. Consequently, their effect on urinary mycotoxin concentration could not be analyzed. Foods that pregnant women commonly consumed in the previous day included spices and condiments (99%), oils and fats (99%), small fish (69%), sugary foods (66%), and hot beverages like tea (52%). Less commonly consumed foods by pregnant women included nuts and seeds (9%), beans and peas (31%), and dark green leafy vegetables (32%). The consumption of local stimulants (betel nut/leaf and many chewing tobacco) by pregnant women in the past month was high.

**Table 5:** Direction of crude associations between consumption of certain foods/stimulants and frequently detected mycotoxins in urine samples of pregnant women in rural Habiganj district, Bangladesh (*N* = 443)

Food group / Stimulant	*n*	OTA		*t*CIT		*t*DON	
		ß sign	*p*-value*	ß sign	*p*-value*	ß sign	*p*-value*
Starches (Yes > 15 g, past 24h)	443	*na*	*na*	*na*	*na*	*na*	*na*
Pulses (Yes > 15 g, past 24h)	139	+	0.43	+	0.47	±	1.00
Nuts/seeds (Yes > 15 g, past 24h)	41	+	< 0.001	+	0.77	±	1.00
Dark green leafy vegetables (Yes > 15 g, past 24h)	142	+	0.05	+	0.73	±	1.00
Vitamin A-rich fruits (Yes > 15 g, past 24h)	47	+	0.13	–	0.34	±	1.00
Vitamin A-rich vegetables (Yes > 15 g, past 24h)	27	+	0.86	–	0.91	+	0.04
Vitamin C-rich fruits (Yes > 15 g, past 24h)	171	+	0.46	+	0.42	–	0.07
Vitamin C-rich vegetables (Yes > 15 g, past 24h)	146	–	0.06	+	0.95	±	1.00
Eggs (Yes > 15 g, past 24h)	75	–	0.03	+	0.33	–	0.23
Organ meat (Yes > 15 g, past 24h)	12	+	0.68	–	0.20	±	1.00
Small fish (Yes > 15 g)	306	+	0.38	+	0.18	–	0.06
Large fish/seafood (Yes > 15 g, past 24h)	219	–	0.04	–	0.34	±	1.00
Flesh foods (Yes > 15 g, past 24h)	66	+	0.80	–	0.02	±	1.00
Dairy (Yes > 15 g, past 24h)	129	–	0.61	–	0.14	±	1.00
Edible oil (Yes > 15 g, past 24h)	437	–	0.05	–	0.18	+	0.67
Sugary foods (Yes > 15 g, past 24h)	292	–	0.01	+	0.27	+	0.08
Condiments/spices (Yes > 15 g, past 24h)	440	–	0.45	–	0.85	–	0.70
Tea or coffee** (Yes > 15 g, past 24h)	232	–	0.01	–	0.95	+	0.06
Betel leaf (*Paan*) (Yes, past 4 weeks)	258	+	0.03	+	0.001	+	0.07
Betelnut (Yes, past 4 weeks)	263	+	0.07	+	0.03	+	0.04
Betelnut and *Paan* (Yes, past 4 weeks)	257	+	0.04	+	0.01	+	0.07
Chewing obacco (*Jorda*) (Yes, past 4 weeks)	193	+	0.01	+	0.02	+	< 0.001

ß sign – the direction of association (positive ( +) or negative (–)) from beta-coefficients; * *p*-values from crude median regression analyses using bootstrapping over 1000 replications; *n* number of consumers, *na*—not analyzed; *t*CIT- total CIT (CIT + HO-CIT); *t*DON—total DON (DON + DON-Glucuronides); **In the study region, tea is consumed much more frequently than coffee, ± means no change in coefficient; h-hours

The regression models showed significant positive associations between urine OTA concentration and intake of more than 15 g of nuts (*p* < 0.0001) and the intake of the following local stimulants in the past month: betel leaf (*Paan)* (*p* = 0.03), betel nut and betel leaf (*p* = 0.04), and chewing tobacco(*Jorda*) (*p* = 0.01). There were also significant negative associations between urine OTA concentration and intake of more than 15 g of eggs (*p* = 0.03), large fish or seafood (*p* = 0.04), sugary foods (*p* = 0.01), and tea or coffee (*p* = 0.01). In the study region, tea is consumed much more frequently than coffee.

Apart from the intake of small animal protein, which was negatively associated with total CIT concentration in urine (*p* = 0.02), none of the other food items was significantly associated with urinary CIT concentration. In contrast, intake of any of the following local stimulants was associated with an increase in urinary CIT levels: betel leaf (*Paan)* (*p* = 0.001), betel nut (*p* = 0.03), *Paan* and betel nut (*p* = 0.01), and chewing tobacco (*Jorda)* (*p* = 0.02).

Likewise, of all food items, only the intake of vitamin A-rich vegetables appeared to increase total DON levels in urine (*p* = 0.04) significantly, while intake of betel nut (*p* = 0.04) and chewing tobacco (*p* < 0.0001) were associated with a significant increase in total DON levels.

## Discussion

The finding that only 17 of 447 urine samples (4%) were free from all investigated mycotoxin biomarkers (Table 2) clearly indicates a widespread exposure of pregnant women in this rural community of Bangladesh to mycotoxins. We detected similar mycotoxins as other biomonitoring studies conducted among pregnant women in the Dhaka district (Ali et al. 2015a, 2016c, 2017). However, this is the first study that determines concurrent multiple mycotoxin exposure among pregnant women in Bangladesh. The results establish that concurrent exposure to multiple mycotoxins (63% of samples) represents this population's prevailing exposure pattern (Figs. 1 and 2). This finding is not surprising considering that different mold species may contaminate a given food sample and that molds require similar conditions for toxin production (Frisvad et al. 2006).

OTA and CIT are the most frequently occurring mycotoxins in this population. A previous biomonitoring study among urban and rural residents in the Rajshahi district of Bangladesh revealed an equally high occurrence of both OTA and CIT biomarkers (> 60%) (Ali et al. 2016a). These are two major hazardous mycotoxins produced mainly by certain *Aspergillus* and *Penicillium* species, frequently contaminating foods and feed under poor post-harvest handling and storage conditions (Alshannaq and Yu 2017; Frisvad et al. 2006). Poor transportation practices, improper drying, improper shelling, poor curing techniques, bare floor storage, and very humid storage conditions are some of the post-harvest and storage conditions that facilitate storage mold contamination and mycotoxin production (Codex Alimentarius 2016). In Bangladesh, rice harvested in one season is usually stored and consumed until a new harvest becomes available.

The results on multi-mycotoxin biomarker analysis indicate a relatively low exposure of the study population to DON (6%), FB_1_ (< 1%), and ZEN (< 1%), toxins produced mainly by *Fusarium* species which contaminate crops such as maize, wheat, and barley in the field (Alshannaq and Yu 2017; Frisvad et al. 2006). However, it must be considered that the limited sensitivity of the analytical method resulted in an overall low number of detects. Repeated cultivation, late planting, poor field hygiene, heavy planting density, and poor water management are some of the pre-harvest or field conditions that may favor mycotoxin contamination (Codex Alimentarius 2016). In a previous biomonitoring survey among 54 pregnant women in the Dhaka district of Bangladesh, no DOM-1 was detected either. At the same time, 52% of the samples were positive for DON (Ali et al. 2015a), which can be explained by the better sensitivity of the immunoaffinity column (IAC)-based single analyte method used by Ali et al. 2015a. Apart from that, the difference in DON occurrence levels may be partly related to different levels of food contamination and food habits in the two study areas. Although DON has previously been detected at moderate levels in maize samples in Bangladesh (Dawlatana et al. 2002), maize is not an important staple in the study area. In general, the low occurrence rates for DON and other trichothecenes in Bangladesh, compared to studies elsewhere (Ali et al. 2016b; Gerding et al. 2015; Warensjö Lemming et al. 2020), are in good agreement with the Bangladeshi nutritional habits of low wheat and maize consumption. Due to the low occurrence of DON in Bangladesh, the applied management of left-censored data according to EFSA might lead to an overestimation of DON exposure, especially in the UB scenario.

In general, our mycotoxin occurrence results are consistent with the results of an earlier comparative study of urinary mycotoxin occurrence patterns (Gerding et al. 2015), which found that 83 of 95 (87%) samples from adult Bangladeshi volunteers were positive for mycotoxin biomarkers, compared to 80% of samples from Germany and 68% from Haiti. Overall, the urines from Bangladesh had high detection rates for OTA and CIT metabolites, while AFM_1_ and FB_1_ were only occasionally detected, and DON and its metabolites were absent from Bangladeshi samples. Apart from food habits, poor agricultural practices contribute to mold contamination of crops and subsequent mycotoxin production. Thus, the frequent high detection of OTA and CIT in this rural community likely indicates a lack of proper post-harvest and storage practices among households. Not surprisingly, a recent survey among the rural households, to which the pregnant women in our study belong, confirmed generally insufficient knowledge of the conditions that favor mold contamination of crops and the measures for prevention at all stages of crop production and storage (Kyei et al. 2021). This lack of knowledge likely contributed to contamination and the frequent detection of mycotoxins in the investigated urine samples.

Estimating the mean probable dietary intake from the concentrations of the most frequently occurring urinary mycotoxins among the pregnant women in our study, we found the values for OTA up to 25 times higher than the previous health-based guidance value of 17.1 ng/kg bw (EFSA 2006). However, in their recent opinion on OTA, the EFSA concluded that based on new data, it is inappropriate to establish a health-based guidance value, and a margin of exposure approach was applied (EFSA 2020). For this reason, MOE values were also calculated, and the vast majority of samples had MOE values far below 10,000 (Fig. 3), indicating a high health concern for the carcinogenic effects of OTA in this population. We also compared the mean estimated probable dietary intake for CIT under various substitution scenarios to the established health guidance level of no concern for nephrotoxicity (200 ng/kg bw) (EFSA 2012) and found that between a fifth and a third of the pregnant women had a dietary intake of CIT at levels of public health concern. Given experimental evidence of transplacental transfer of mycotoxins such as AF, OTA, CIT, DON, and ZEN to the developing fetus (Goyarts et al. 2007; Nielsen et al. 2011; Partanen et al. 2010; Reddy et al. 1982; Warth et al. 2019; Woo et al. 2012), exposure of pregnant women to these mycotoxins also represents a potential risk to maternal, fetal and child health, requiring further investigations to ascertain associations and mechanisms.

When examining possible dietary sources of the most frequently occurring mycotoxins using regression models, we found that intake of certain foods and local stimulants (betel nut, betel leaf, and chewing tobacco) was associated with urinary mycotoxin biomarker concentrations (Table 4). For example, intake of nuts and seeds (mainly groundnuts in our study area) and local stimulants was associated with higher urinary OTA concentration, while intake of eggs, large fish, or other seafood, but also of sugary foods and tea was associated with lower OTA concentrations. While associations point to certain foods/stimulants that may be contaminated, the proportion of women consuming such foods/stimulants determines whether that consumption can explain the detected mycotoxin occurrence in urine. For example, although we found that nut consumption was positively associated with urinary OTA levels, less than 10% of women consumed 15 g of nuts or seeds in the past day, which suggests this cannot be the primary dietary source. Local stimulant consumption was associated with higher urinary concentrations of OTA, CIT, and DON, and the majority of women took betel nut or leaf, and many women took chewing tobacco in the past month (Table 4), suggesting that these stimulants may be responsible for the high urinary concentrations identified in many pregnant women. Notably, OTA and CIT, the most frequently detected mycotoxins, have been reported in rice in Asia, the primary staple food in the sub-region (Ali et al. 2018; Reddy et al. 2009). However, as rice was consumed daily by all the women in our study, it was impossible to analyze its association with urinary mycotoxin concentrations.

A previous study in Bangladesh detected aflatoxin levels above US maximum regulatory levels in eight commonly ingested food commodities, including rice, lentils, wheat flour, dates, betelnut, and groundnut (Roy et al. 2013). AFM_1_ was, however, detected in only a few urine samples in our pregnant cohort, suggesting that very high aflatoxin exposure does not frequently occur in this community. Still, given that AFM_1_ has been detected in greater frequency in other studies in Bangladesh (Ali et al. 2017), this low occurrence may be a limitation of using a urine biomarker that only reflects acute exposures, although the LOD/LOQ obtained with the used 'dilute and shoot' approach is higher compared to other studies applying an additional sample cleanup step (Ali et al. 2017; Šarkanj et al. 2018). Consequently, to determine whether aflatoxin exposure is really low in our study population, measurement of aflatoxin B_1_-lysine adduct in blood samples will be carried out in a follow-up project. This long-term biomarker based on the formation of aflatoxin-B_1_-albumin allows for accurate and sensitive quantification of AFB_1_ uptake over the past two months.

The present study is unique due to the large study sample of pregnant women (*n* = 447) with paired data from the FAARM food consumption survey and the multiple urinary mycotoxin biomarker analysis. The applied method is based on a dilute and shoot approach, covering a broad spectrum of compounds but focusing on the previously detected highly relevant mycotoxins OTA and CIT / HO-CIT. For other analytes, a moderate sensitivity was accepted as no more sensitive methods with comparable efficiency were available at the time of sample analysis (Schmidt et al. 2021a). As a consequence, the LODs of some mycotoxins were comparably high, allowing only the detection of very high exposure scenarios but not the baseline level of mycotoxin uptake present in Bangladesh.

While urine is convenient for population-based biomonitoring studies due to the non-invasive sampling involved, the daily variation in urine composition and excretion rate among individuals adds some difficulties. We adjusted for inter-individual variations in urine volume by correcting urine mycotoxin concentrations for urine density and urine creatinine before dietary exposure estimation. Despite the inherent challenges in assessing diets accurately given misreporting and misclassification biases, we found evidence that reported intake of certain foods and stimulants was linked to urinary mycotoxin concentrations. However, our dietary assessment was not comprehensive. For example, spices were not covered in our dietary survey but are also a common source of high OTA contamination (Kabak and Dobson 2017). Sampling common foods and stimulants, as well as spices, from our study population for direct mycotoxin contamination analysis will be valuable in ascertaining the sources of dietary mycotoxin contamination in this population. Our study was conducted among pregnant women from rural households in Habiganj district who participated in the FAARM trial, and thus the conclusions cannot necessarily be generalized to other areas. However, our results were similar to those from previous biomonitoring studies in Bangladesh, suggesting that our study population is typical for rural settings in Bangladesh, and findings may, thus, also apply to similar settings.

Overall, the following conclusions can be drawn from our study:

- Exposure to multiple mycotoxins during early pregnancy is widespread in this rural community and represents a potential health risk for mothers and babies.
- OTA alone or in combination with citrinin, produced by post-harvest or storage molds, are ubiquitous food contaminants in this community.
- FB_1_, ZEN, DON, and other trichothecenes, produced by pre-harvest or field molds, are less common contaminants of foods consumed by pregnant women in this community.
- The local stimulants betel nut, betel leaf, and chewing tobacco may be possible sources of OTA, CIT, and DON exposure among pregnant women in this community.

Although mycotoxins cannot be completely eliminated from food or feed supplies, their levels can be substantially reduced by promoting good agricultural practices, safe food habits and proper storage. This is an important challenge for agricultural extension work. Future research should explore the role of concurrent exposure to multiple mycotoxins for the development of adverse health outcomes in mothers, newborns, and children.

## Supplementary Information

Below is the link to the electronic supplementary material.

Supplementary file1 (XLSX 13 KB)

## Data Availability

The data presented in this study are available upon reasonable request from the corresponding author. The data are not publicly available due to privacy restrictions.
